# Association of Membranous Basal Cell Adenoma and Basal Cell Adenocarcinoma With Brooke-Spiegler Syndrome

**DOI:** 10.7759/cureus.68819

**Published:** 2024-09-06

**Authors:** Lexi Goehring, Debby Rampisela, Jordan L Pleitz

**Affiliations:** 1 Otolaryngology, Texas A&M College of Medicine, Temple, USA; 2 Pathology, Baylor Scott and White Health - Temple, Temple, USA; 3 Otolaryngology and Head and Neck Surgery, Baylor Scott and White Health - Round Rock, Round Rock, USA

**Keywords:** basal cell adenocarcinoma, basal cell adenoma, brooke spiegler syndrome, cyld gene, salivary gland tumors

## Abstract

Basal cell adenoma (BCA) and basal cell adenocarcinoma (BCAC) are uncommon basaloid biphasic salivary gland tumors composed of basal and ductal cells. BCAC is differentiated from BCA by the presence of invasion in BCAC. In this paper, an 82-year-old woman presented with a palpable 3 cm immobile mass in her right parotid gland. A computed tomography (CT) scan showed two separate right parotid masses. She underwent a right parotidectomy, and the pathology showed multiple membranous BCAs and BCAC, which were highly suspicious for Brooke-Spiegler syndrome (BSS). This paper discusses BCA, BCAC, and their relationship with BSS.

## Introduction

Basal cell adenoma (BCA) and basal cell adenocarcinoma (BCAC) were defined as specific entities by the World Health Organization (WHO) in 1991 [1]. BCA is an infrequent benign tumor, comprising 3-4% of salivary gland tumors, whereas BCAC is a rare malignant tumor, accounting for 2.9% of salivary gland tumors [1]. BCA and BCAC share similar cytomorphology; however, BCAC has infiltrative features. BCAC is generally regarded as a low-grade malignancy; however, rare high-grade cases have been reported. Histologically, BCA has a variety of cellular arrangements, including trabecular, tubular, solid, and membranous patterns [2]. The membranous type often shows a multinodular growth pattern and has been associated with dermal cylindromas or trichoepithelioma [3]. Multiple membranous basal cell lesions in the salivary gland with cutaneous lesions are highly suspicious for Brooke-Spiegler syndrome (BSS). BSS, also known as CYLD cutaneous syndrome (CCS), is a rare autosomal dominant condition first described separately in 1892 and 1899 [4]. The disease manifests as multiple skin tumors, including cylindromas, spiradenomas, and trichoepitheliomas, 90% involving the head and neck. A biopsy of skin lesions with a genetic test can confirm the diagnosis of BSS [4-5].

## Case presentation

An 82-year-old woman presented with a six-month history of a slowly enlarging right pre-auricular mass. She reported some discomfort associated with swelling but denied pain, drainage, facial weakness/numbness, fevers, chills, or unanticipated weight loss. Her significant medical history included chronic otitis media post right canal-wall-down tympanomastoidectomy, hypothyroidism, hypercholesterolemia currently under treatment, and osteoarthritis of the hands. She had a remote tobacco abuse (an average of 1.5 packs per day) and quit about 28 years ago. She had an excision of a 0.3 cm papule from her left ala nose approximately 10 years ago and several cryotherapy treatments of multiple scaly lesions presumed as actinic keratosis and seborrheic keratosis on her bilateral ears, arms, and back. Dermatologists did the evaluations of the skin lesions and the procedures.

On physical examination, there was a firm, immobile 3 cm mass involving the anterosuperior right parotid gland. There was no appreciable lymphadenopathy or tenderness to palpation within the neck. A CT scan with contrast revealed two separate lesions involving both the anterior gland and parotid tail.

**Figure 1:**
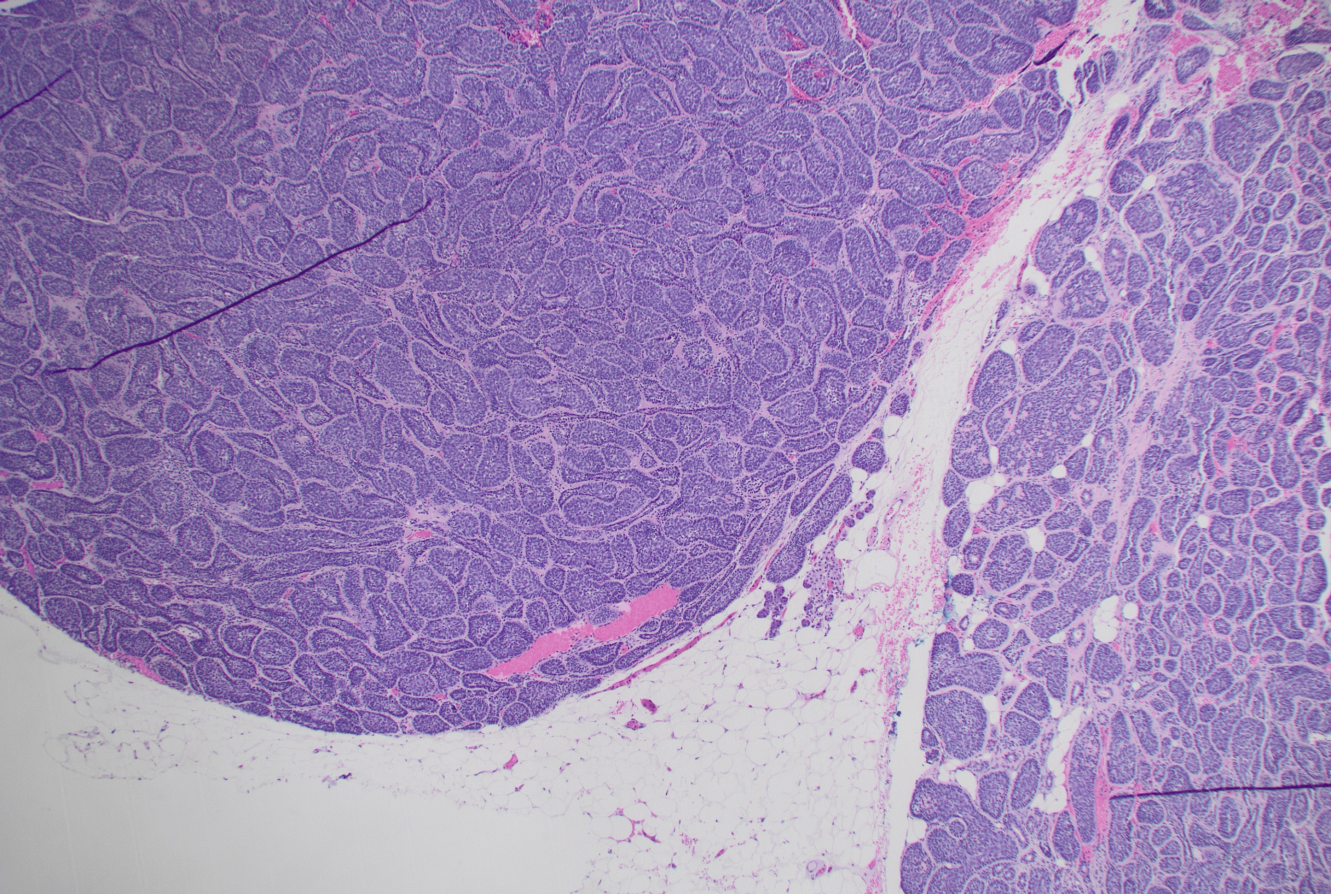
Membranous basal cell adenoma, well-circumscribed with nests of basaloid cells with hyaline basement membrane-like material, H&E stain, 40x.

**Figure 2:**
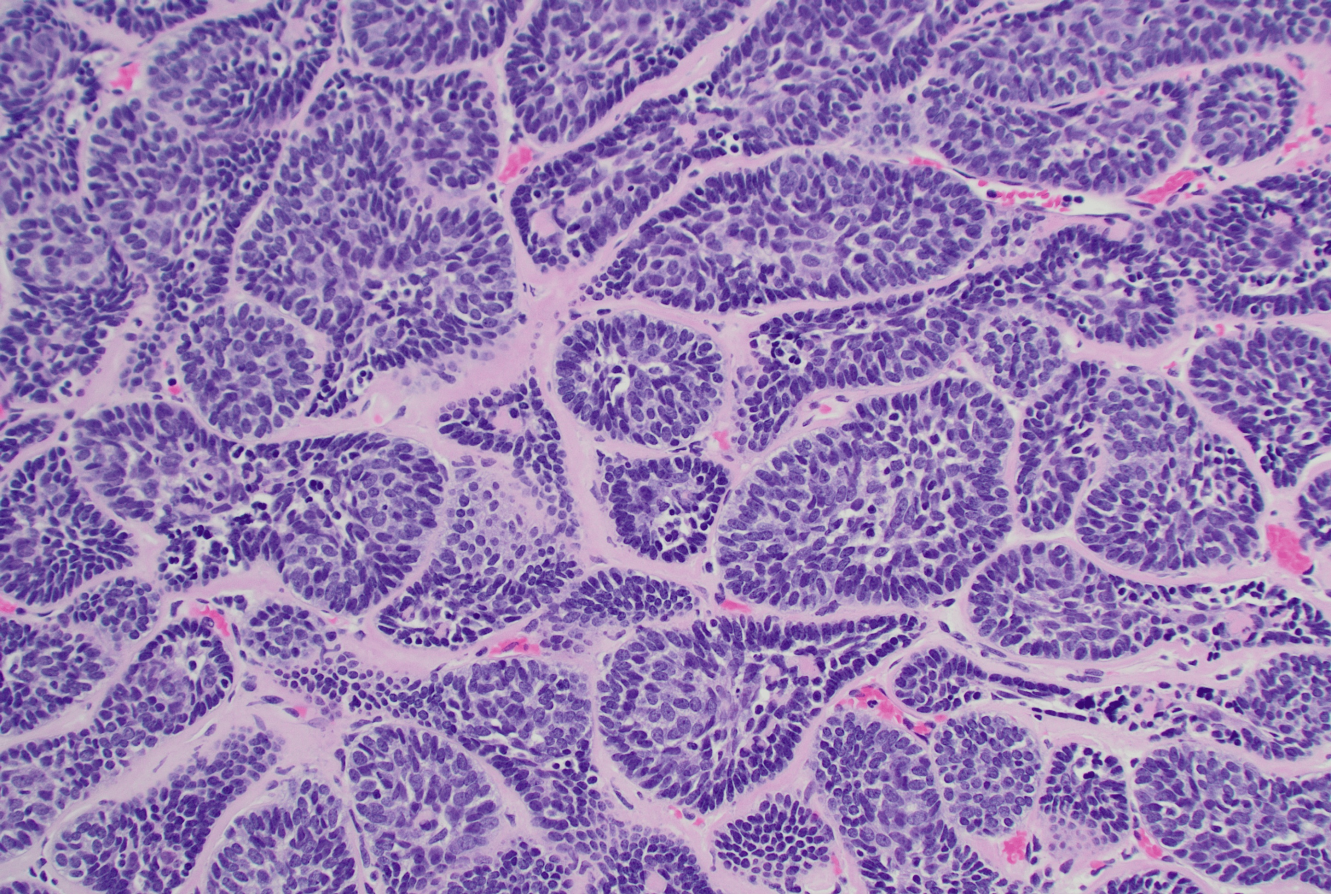
Membranous basal cell adenoma, a jigsaw puzzle-like pattern of basaloid nests surrounded by thick basement membrane-like material, H&E stain, 100x.

**Figure 3:**
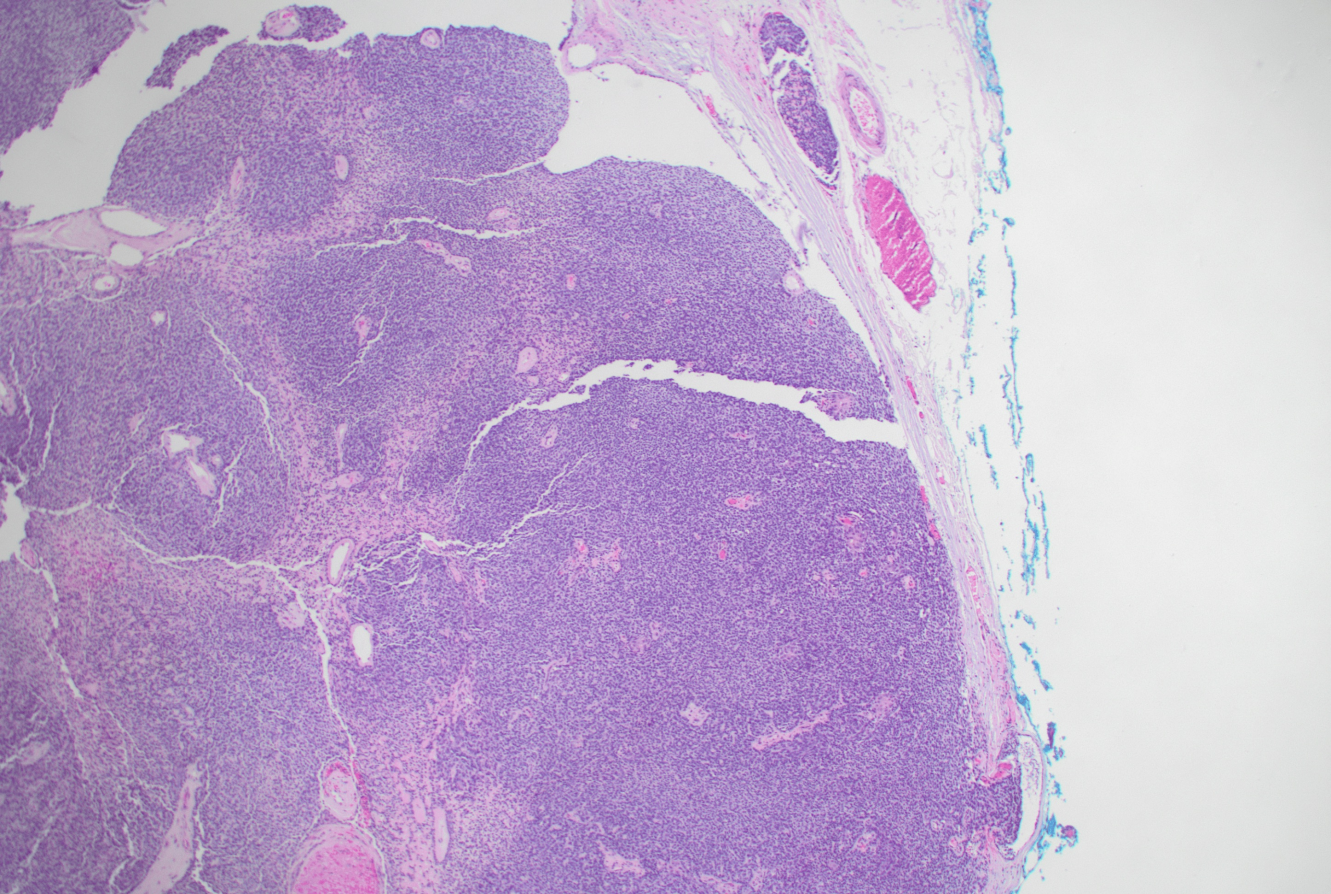
Basal cell adenocarcinoma, a solid pattern of basaloid cells with lymphovascular invasion, H&E stain, 40x.

**Figure 4:**
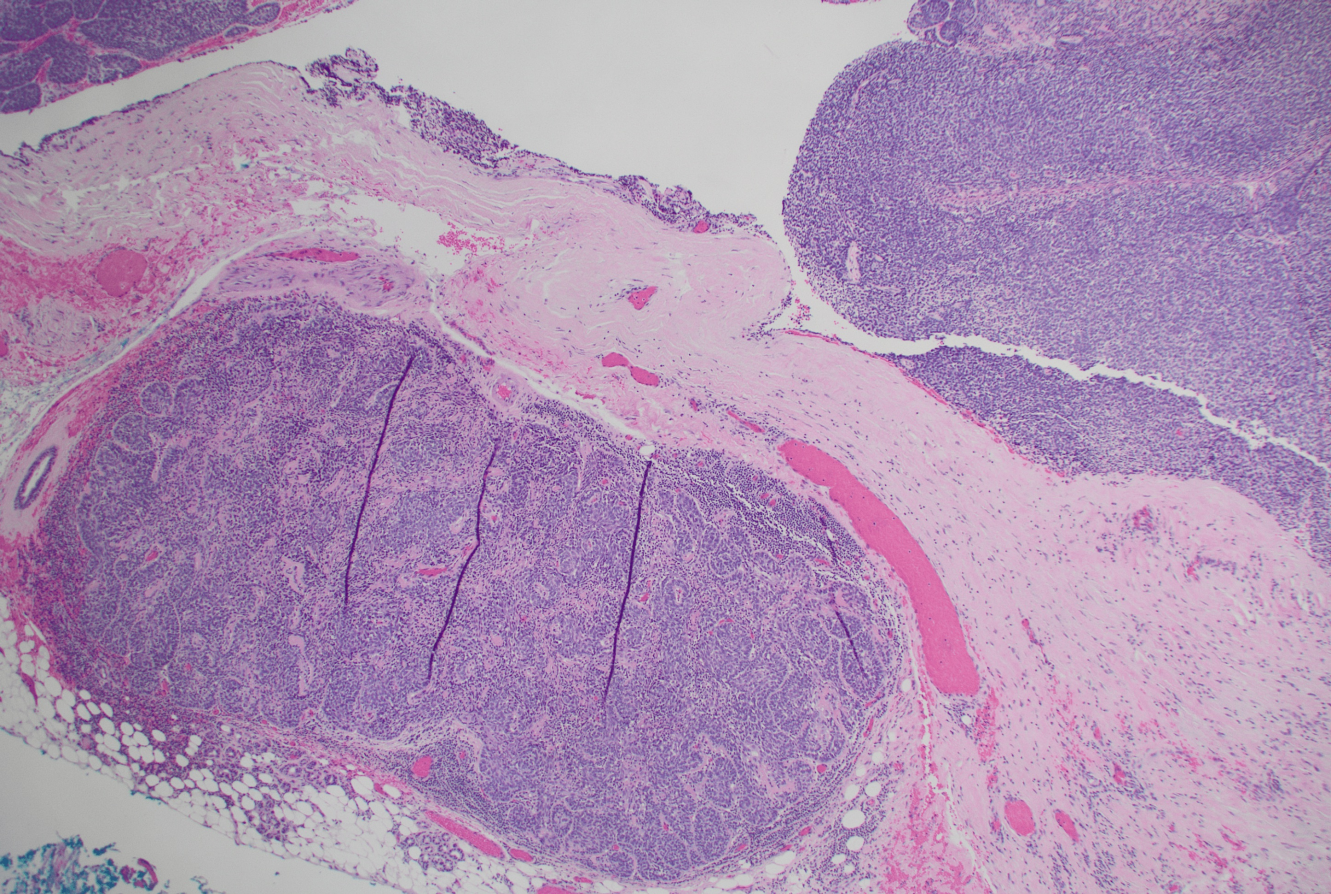
Basal cell adenocarcinoma with invasion into the adipose tissue, H&E stain, 40x. Inset: basaloid cells with mitotic figure, H&E stain, 400x.

The patient elected to undergo a total right parotidectomy. Surgical pathology demonstrated 0.8 and 1.3 cm membranous BCAs and a 2.6 cm low-grade BCAC (Figures 1-4). The BCAs and BCAC showed basaloid cells with scattered ducts with a jigsaw pattern surrounded by hyaline basement membrane-like material. This jigsaw pattern was diffuse in BCAs but focal in BCAC. The BCAC had a predominant solid pattern with scattered mitoses and showed invasion into the surrounding soft tissue and lymphovascular invasion. Immunostains of BCA and BCAC illustrated a biphasic pattern with basal cells of a myoepithelial phenotype showing diffuse positivity for p63 and patchy positivity for S100 and smooth muscle actin (SMA). Ductal cells displayed diffuse positivity for CK7 and patchy positivity for CD117. The tumor showed no nuclear staining for beta-catenin.

The patient had multiple scaly lesions suggestive of actinic keratosis and seborrheic keratosis, but there were no obvious neoplastic skin lesions. The patient refused a more thorough dermatology examination and genetic testing for BSS. The previous biopsy of the nose's skin nodule was re-reviewed and showed trichodiscoma (Figure 6).

## Discussion

Salivary gland tumors encompass heterogeneous groups of neoplasms with at least 36 different tumor types [6]. BCA and BCAC are two types of basaloid tumors of the salivary gland, which predominantly occur in major salivary glands, particularly the parotid gland. BCA and BCAC should be differentiated from other biphasic basaloid tumors of the salivary glands, such as cellular pleomorphic adenoma, adenoid cystic carcinoma, and epithelial-myoepithelial carcinoma. In most cases, surgical excision is needed for a definitive diagnosis due to the limitation of the fine needle aspiration or needle core biopsy to differentiate these tumors, which can have similar cytomorphology [7].

BCA is a benign biphasic basaloid neoplasm consisting of basaloid cells with a prominent basal cell layer and ductal (luminal) cells with basement membrane-like material [2]. BCA predominantly occurs in the parotid gland and more commonly affects women in the fifth decade. The majority of BCA cases present with a single movable solid nodule. Although uncommon, a cystic nodule can also be encountered. BCA is well-circumscribed and often encapsulated. By histomorphology, BCA is divided into trabecular, tubular, solid, and membranous variants. In particular, recognizing the membranous variant is essential because of its propensity to recur [2-3]. The recurrence, observed in nearly 25% of cases, is attributed to the multinodular growth pattern of the tumor [2].

BCAC is a rare malignant neoplasm opposite of BCA, defined by its invasive features [1,8]. Generally, BCAC is considered a malignancy that is locally aggressive but rarely metastasizes. However, cases of high-grade BCAC have been reported. BCAC shares similar morphologic patterns with BCA, including trabecular, tubular, solid, and membranous patterns. Solid pattern is more commonly seen in BCAC. BCAC can arise from preexisting BCA or arise de novo. BCAC has infiltrative growth patterns such as invasion into the surrounding tissue, intravascular, and perineural invasion. The presence of necrosis, high mitotic activity, or marked nuclear pleomorphism is helpful for the diagnosis of high-grade BCAC.

Most cases of BCA and BCAC show similar clinical findings, imaging, and gross appearances of the tumors [1]. Small numbers of BCAC cases show pain/tenderness, adhesion to surrounding tissue, facial palsy, hemorrhage, or necrosis. BCA and BCAC are best diagnosed using routine hematoxylin and eosin sections, particularly in the resection specimens.

BCA and BCAC share similar molecular findings, more commonly CTNNB1 and CYLD1 alterations [8]. In BCA, CTNNB1 alterations are often present in tubulotrabecular BCA, while CYLD1 alterations are often present in membranous BCA. It is worth noting that gene alterations are less commonly seen in BCAC compared to BCA. CTNNB1 codes for beta-catenin, correlating to tumor cells’ nuclear expression with beta-catenin immunohistochemistry. CYLD gene is more commonly discussed in association with Brooke-Spiegler syndrome (BSS).

BSS/CYLD cutaneous syndrome is an inherited autosomal dominant disease with numerous associated cutaneous adnexal tumors, including cylindroma, spiradenoma, and trichoepithelioma. BSS phenotypic variants include multiple familial trichoepithelioma-1 (MFT1) and familial cylindromatosis (FC) [9]. BCA, particularly membranous BCA and BCAC, occur in BSS in a minority of cases [5]. Conversely, CYLD alterations in BCA or BCAC occur mainly in cases with no syndromic conditions [8]. The definitive diagnosis of BSS is based on the combination of clinical and pathologic findings, including a clinical history of multiple skin neoplasms at a young age with a family history of similar neoplasms, biopsy findings of cylindroma, spiradenoma, or trichoepithelioma, the association with BCA or BCAC of the major or minor salivary glands, and the presence of a germline mutation in CYLD by genetic testing.

Our case was highly suspicious for BSS syndrome. Unfortunately, the diagnosis cannot be confirmed because the patient refused further investigation. It is also worth noting that the patient had a solitary tricodiscoma, which was not a syndromic disease. If there are multiple trichodiscomas Birt-Hogg- Dubé syndrome (BHD) should be considered. BHD is an autosomal dominant genodermatosis with an aberration in the folliculin (FLCN) gene. The patients usually present with multiple skin lesions, particularly trichodiscomas, fibrofolliculomas, and acrochordons. There is an increased susceptibility in BHD patients to develop renal cell carcinoma, spontaneous pneumothorax, and lung cysts [10].

The primary treatment for BCA, BCAC, and tumors in BSS is complete surgical excision. The presence of multiple basaloid parotid tumors should raise the possibility of BSS; therefore, a thorough clinical history with physical examination in conjunction with tissue sampling and genetic consultation/testing should be done.

## Conclusions

BCA and BCAC are uncommon basal cell salivary gland tumors diagnosed through histopathology. In cases of multiple basal cell tumors in salivary glands, BSS should be suspected. To confirm the diagnosis of BSS, assess clinical findings with skin biopsies or genetic testing for CYLD mutation. Surgical excision is the primary treatment for BCA and BCAC, with a generally favorable prognosis, as BCA is benign and BCAC is typically low-grade.

## References

[1] Terada T, Ryo K, Masaaki H, Kurisu Y, Hiroko K, Hirose Y (2021). Basal cell adenocarcinoma of the parotid gland: comparison with basal cell adenoma for preoperative diagnosis. Auris Nasus Larynx.

[2] Ben Abdeljelil N, Masmoudi M, Thabet W, Bellalah A, Chebil E, Hasnaoui M, Mighri K (2024). Basal cell adenoma of the parotid gland: a rare entity. Ear Nose Throat J.

[3] Jeddy N, Prasannamoorthy L, Thavarajah R, Radhika T, Ramachandran A (2017). Membranous basal cell adenoma - a rare entity in an unusual location. J Clin Diagn Res.

[4] Mohiuddin W, Laun J, Cruse W: Brooke-Spiegler Syndrome (2018). Brooke-Spiegler syndrome. Eplasty.

[5] Jungehülsing M, Wagner M, Damm M (1999). Turban tumour with involvement of the parotid gland. J Laryngol Otol.

[6] Rito M, Esteves S, Fonseca I (2022). Basal cell adenoma and basal cell adenocarcinoma: a 50-year experience from a single institution. Head Neck Pathol.

[7] Song IH, Song JS, Sung CO (2015). Accuracy of core needle biopsy versus fine needle aspiration cytology for diagnosing salivary gland tumors. J Pathol Transl Med.

[8] Robinson RA (2021). Basal Cell adenoma and basal cell adenocarcinoma. Surg Pathol Clin.

[9] Kazakov DV (2016). Brooke-Spiegler syndrome and phenotypic variants: an update. Head Neck Pathol.

[10] Tong Y, Schneider JA, Coda AB, Hata TR, Cohen PR (2018). Birt-Hogg-Dubé syndrome: a review of dermatological manifestations and other symptoms. Am J Clin Dermatol.

